# Supplementary material for: Detection of Mycobacterium tuberculosis using Gene Xpert-MTB/RIF assay among tuberculosis suspected patients at Mizan-Tepi university teaching hospital, southwest Ethiopia: An institution based cross-sectional study
Source: PLoS One. 2022 Nov 23;17(11):e0277536. doi: 10.1371/journal.pone.0277536 (PMC9683564; doi:10.1371/journal.pone.0277536)
Supplement: Attachment — Submitted filename: Response to Reviewers.docx [file pone.0277536.s002.docx]

**Response to reviewers**

| Editor comment | Author response | Page number |
| --- | --- | --- |
| 1. Please ensure that your manuscript meets PLOS ONE's style requirements, including those for file naming. | Authors incorporated and highlight each requirement in revised manuscript, for example, heading, font size, use italics and text formatting, figures and table citation and etc. |  |
| 2. Thank you for stating the following financial disclosure:   "The funders had no role in study design, data collection and analysis, decision to publish, or preparation of the manuscript."  At this time, please address the following queries:  a) Please clarify the sources of funding (financial or material support) for your study. List the grants or organizations that supported your study, including funding received from your institution.  b) State what role the funders took in the study. If the funders had no role in your study, please state: “The funders had no role in study design, data collection and analysis, decision to publish, or preparation of the manuscript.” c) If any authors received a salary from any of your funders, please state which authors and which funders. d) If you did not receive any funding for this study, please state: “The authors received no specific funding for this work.” | We mentioned in the revised manuscript that **“The authors received no specific funding for this work.”** (Please see Declaration section). | 17 |
| 3. In your Data Availability statement, you have not specified where the minimal data set underlying the results described in your manuscript can be found. PLOS defines a study's minimal data set as the underlying data used to reach the conclusions drawn in the manuscript and any additional data required to replicate the reported study findings in their entirety. All PLOS journals require that the minimal data set be made fully available..  "Upon re-submitting your revised manuscript, please upload your study’s minimal underlying data set as either Supporting Information files or to a stable, public repository and include the relevant URLs, DOIs, or accession numbers within your revised cover letter.  Important: If there are ethical or legal restrictions to sharing your data publicly, please explain these restrictions in detail. We will update your Data Availability statement to reflect the information you provide in your cover letter. | Authors mentioned in the revised manuscript that “The data supporting our findings were provided as a part of a manuscript”. (Please see declaration section). | 16 |
| 4. PLOS requires an ORCID iD for the corresponding author in Editorial Manager on papers submitted after December 6th, 2016. Please ensure that you have an ORCID iD and that it is validated in Editorial Manager. To do this, go to ‘Update my Information’ (in the upper left-hand corner of the main menu), and click on the Fetch/Validate link next to the ORCID field. This will take you to the ORCID site and allow you to create a new iD or authenticate a pre-existing iD in Editorial Manager. Please see the following video for instructions on linking an ORCID iD to your Editorial Manager account: <https://www.youtube.com/watch?v=_xcclfuvtxQ> | I had an ORCID ID and I validated it in Editorial Manager |  |
| 5. Please amend the manuscript submission data (via Edit Submission) to include author Murtii Teressa. | We amended it |  |
| Review Comments to the Author | **Author response** | **Page number** |
| Reviewer #1:  This is observational study looking in proportion of patients with documented TB among patients with presumptive TB, and risk factors associated with the detection of TB in this group.  Study design: The problem is that this is a very biased population and the results do therefore not reflect the TB prevalence, but rather the positivity rate of GeneXpert in patients in whom a sample could be retrieved. All EPTB studied had a sample, only patients who can produce sputum are considered. It is unclear how the patients were included, based on a sample that was produced, are based on symptoms. For the latter it is unclear which symptoms were considered. | We tried to revise some unclear ideas. In detail, to make it uniform, we included those patients with age group of ≥ 5 years. In addition, we considered clinical signs and symptoms of TB such as night sweat, loss of appetite, loss of weight, fever, and cough which lasts at least for 2 weeks for enrolment of patients (Please see method section). | 5 |
| Sample processing: as Xpert MTB/RIF is a well-known method there is no need to put the laboratory procedures in such detail. Why the RIF resistance rate was not reported? In the discussion you say that it is insignificant, still it is OK to report it in the result. | We tried to shorten the detail procedures and we included the report of RIF resistance rate in the revised manuscript (Please see in method and Result section). | 7 &10 |
| Results: English: several sections need heavy language editing, but especially the results. Avoid using local currency if you mean to publish in international journals or add the equivalent in Dollars or Euro. | We tried revised some language copy editing errors and we changed the local currency in Dollars in the revised manuscript (Please see in method and Result section). | 5 to 12 |
| Table 2: it is surprising that only 7% of patients were vaccinated with BCG. How was this defined? | It is not 7%, instead it is 12.1%. Sorry for the editorial errors (Please see table-4 in the Result section). | 12 |
| Table 1 and 4 can be combined, as can table 2 and 5. | We merged table 2 and 5 to form table 4 | 12 |
| Why are students selected as the reference population in table 4; The OR are not correct. I did a check: if you compare farmers with students the OR = 0.83. If you compare farmers with all others the OR = 0.78. Assume that the OR were correct, then the reporting is not correct: farmers are less likely to have a positive TB result, if their a OR = 0.37. | We accept the comments and we made analysis on the variable occupation and we revised it in the revised manuscript. In addition, we selected govt employee as a reference population because we expected that govt employee have good awareness on TB transmission and prevention which resulted in low proportion of TB positive rate among this population groups. | 10 and table 3 |
| Reviewer #2: General comment:  This study investigated the prevalence of tuberculosis by geneXpert TB detection system. I agree with the authors that it is very important to determine the prevalence the diseases at every level and different risk groups to monitor the national program’s performance to attain the 2035 goal in Ethiopia. It is institutional based study with its draw backs that it cannot be extrapolated to the general population in the study area. I suggest authors to address the flowing points in order to improve the manuscript. Major and Compulsory Revisions: | Generally, we tried to revise point-by- point throughout the manuscript based on your comments and were incorporated in the revised manuscript. In detail, addition or removal of words, sentences, clarifying unclear ideas, comments on rephrasing and etc were incorporated and **highlighted** in the revised manuscript. However, if it need additional improvement authors are ready to do it. |  |
| 1. The authors need language support in the writing. | We tried to revise some language copy editing errors and **highlighted** in the revised manuscript. | 5 to 12 and throughout the manuscript |
| 2. Title: The title looks that the whole work of the article is dealing with infection with *M. tuberculosis* but not clinical TB. The authors need to make clear distinction between infection with *M. tuberculosis* and development of TB disease. | The author tried to investigate positivity of *M. tuberculosis* among clinically TB suspected patients. It is obvious, if the patients are first clinically suspected as to have TB and then if *M. tuberculosis* was detected, this patient has clinical TB. Therefore, authors tried to revise based on the comments in the revised manuscript. | Title page and the whole body |
| 3. Abstract: Conclusion: The authors concluded that their finding reveals increased prevalence of *M. tuberculosis* infection. However, there was no supporting data trends that suggests the increased prevalence the disease overtime. | The authors tried to revise based on the comments in the revised manuscript. | 1 and 2 |
| 4. Introduction: The first sentence:” Tuberculosis, or TB, is an airborne infectious disease caused by *Mycobacterium tuberculosis* and is the leading cause of ill health and related deaths worldwide.” Is not true as it is against the first sentence of the second paragraph and currently available data. | The authors revised the raised comments in the revised manuscript. | 3 |
| 5. Study area and period: Authors indicated that “Of the total annual patient visits, approximately 1080 people were visit a TB clinic.” But there was no indication about how many TB clinics are there at MTUH? | The authors revised as ‘Only one TB clinic was available at the hospital, and annually, an average of 610 TB confirmed cases were visit a TB clinic.’ | 5 |
| 6. Laboratory sample collection a. Authors described that “Those patients identified with signs and symptoms of tuberculosis were ordered to bring a single appropriate sample for the diagnosis of TB.” Which signs and symptoms of TB were considered? b. There was no indication how the investigators identified cases that has started anti-TB treatment or not. | Those patients with age group of ≥ 5 years and those with clinical signs and symptoms of TB such as night sweat, loss of appetite, loss of weight, fever, and cough which lasts at least for 2 weeks were considered as presumptive TB cases.  In addition, if the patients come for follow up, we treated these patients as he/she started anti-TB treatment. | 5 |
| 7. Sample processing: Which generations of cartridges were used? | It is good, but some information is not captured by data collectors at the time. Actually, the data collectors are those laboratory professionals working at microbiology laboratory and this work is done in line with the routine work of the microbiology laboratory at the time. Sorry for the moment, and if it is mandatory, I will include it in the next revision. | - |
| 8. Result: The authors described that “A majority of the respondents 164 (38.9%) were farmers”. How did the investigators determine majority? | We accept the comment and revised as ‘Farmers accounted for 164 (38.9%)” | 8 |
| 9. Factors associated with Prevalence of TB: Authors reported that *M. tuberculosis* was detected in 18 (10.8%) of farmers, a proportion, which was 63.0% times greater than other occupations (AOR: 0.370; 95% CI: 0.143, 0.953). The data is wrongly described/presented as in the table the proportion of positive cases were more in groups for instance daily labourers and prisoners. The same wrong conclusion was taken in discussion as well. | We accept the comments and we made analysis on the variable occupation and we revised it in the revised manuscript. In addition, we selected gov’t employee as a reference population because we expected that govt employee have good awareness on TB transmission and prevention which resulted in low proportion of TB positive rate among this population groups. | 10 and Table 3 |
| 10. Discussion a. The authors indicate that “The result of this study was higher when compared with study conducted at Jimma University Medical Center (9.3%) [17], Southwest Ethiopia, Ataye District Hospital (9.0%) [18] and Motta general hospital (8.4%) [19], North Ethiopia”. However, their comparison did not take into account the 95% confidence around the figure (prevalence) reported by the authors. b. They compared their findings with numerous published articles but did not discuss on individual basis why the difference was observed. | Although we didn’t present it in separate (individual) bases, we tried to discuss why the variations were observed between the studies. If we present in individual bases, the discussion may be more extended with repetition of ideas. | 13 |
| 11. Minor Essential Revisions a. Sample size: The use of p= 0.5 is not justified as there are numerous studies from Ethiopia that can be used to calculate the more appropriate size. | To get maximum sample size, p= 0.5 is better and we used it | 6 |
| b. How was malnutrition determined? | Malnutrition was determined with malnutrition anthropometry (MUAC). | 11 |
| c. How was contact with known TB case was defined in terms of frequency and duration? | A close contact is defined as living in the same household or in frequent contact with an individual with pulmonary tuberculosis in the household or other indoor setting for a minimum of three months prior to diagnosis of TB disease of the index case. |  |
| d. Result: Table 3 and figure 1 are repetitive | We deleted the figure 1 | 10 |
| e. Ethical issues: the reference number of the ethical clearance by the competitive IRB should be indicated. | Approval Number/ID: Rf/274/2021 | 16 |
